# Multifaceted Roles of ICP22/ORF63 Proteins in the Life Cycle of Human Herpesviruses

**DOI:** 10.3389/fmicb.2021.668461

**Published:** 2021-06-07

**Authors:** Ying Wu, Qiqi Yang, Mingshu Wang, Shun Chen, Renyong Jia, Qiao Yang, Dekang Zhu, Mafeng Liu, Xinxin Zhao, Shaqiu Zhang, Juan Huang, Xumin Ou, Sai Mao, Qun Gao, Di Sun, Bin Tian, Anchun Cheng

**Affiliations:** ^1^Institute of Preventive Veterinary Medicine, Sichuan Agricultural University, Chengdu, China; ^2^Avian Disease Research Center, College of Veterinary Medicine, Sichuan Agricultural University, Chengdu, China; ^3^Key Laboratory of Animal Disease and Human Health of Sichuan Province, Sichuan Agricultural University, Chengdu, China

**Keywords:** viral life cycle, latent, lytic, reactivation, ICP22, ORF63

## Abstract

Herpesviruses are extremely successful parasites that have evolved over millions of years to develop a variety of mechanisms to coexist with their hosts and to maintain host-to-host transmission and lifelong infection by regulating their life cycles. The life cycle of herpesviruses consists of two phases: lytic infection and latent infection. During lytic infection, active replication and the production of numerous progeny virions occur. Subsequent suppression of the host immune response leads to a lifetime latent infection of the host. During latent infection, the viral genome remains in an inactive state in the host cell to avoid host immune surveillance, but the virus can be reactivated and reenter the lytic cycle. The balance between these two phases of the herpesvirus life cycle is controlled by broad interactions among numerous viral and cellular factors. ICP22/ORF63 proteins are among these factors and are involved in transcription, nuclear budding, latency establishment, and reactivation. In this review, we summarized the various roles and complex mechanisms by which ICP22/ORF63 proteins regulate the life cycle of human herpesviruses and the complex relationships among host and viral factors. Elucidating the role and mechanism of ICP22/ORF63 in virus–host interactions will deepen our understanding of the viral life cycle. In addition, it will also help us to understand the pathogenesis of herpesvirus infections and provide new strategies for combating these infections.

## Introduction

Herpesviruses are large DNA viruses that can infect a wide range of hosts, including almost all vertebrates and even invertebrates (McGeoch et al., 1995; Carter et al., 2007). During infection, herpesviruses enter mainly through the skin, mucous membranes, and nerve tissue of hosts, and herpesvirus infections seriously affect the health of humans and other animals (Davison et al., 2009; Azab et al., 2018; Heldwein, 2018). To date, more than 100 herpesviruses have been discovered and classified into α-, β-, and γ-herpesviruses, on the basis of their molecular and biological characteristics (Whitley, 1996; Bigalke and Heldwein, 2017). The members of each subfamily possess the same virion structure, including a linear double-stranded DNA genome, capsid, tegument, and envelope (Griffin, 2007; Loret et al., 2008; Johnson and Baines, 2011; Heming et al., 2017; Liu et al., 2019; Lv et al., 2019; Yang et al., 2020). The life cycle of herpesviruses consists of two phases: lytic infection and latent infection (Stevens, 1989; Knipe and Cliffe, 2008; Dupont and Reeves, 2016; Riaz et al., 2017; Münz, 2019; Cohen, 2020). During lytic infection, shortly after entry into a susceptible cell, virions progress through uncoating, gene transcription, DNA replication, protein translation, assembly, egress, release, etc., to produce progeny virions (Glaunsinger, 2015; Heldwein, 2016; Sathiyamoorthy et al., 2017; Lv et al., 2019; Yan et al., 2019; Cohen, 2020). However, active replication of herpesviruses tends to be limited by the host immune system, leading to lifetime latent infection of the host. During latent infection, the viral genome remains in an inactive state in the host cell to avoid host immune surveillance, but the virus can reactivate and reenter the lytic cycle (Grinde, 2013; Retamal-Diaz et al., 2015; Suazo et al., 2015; Adler et al., 2017; Laing et al., 2018; Tognarelli et al., 2019; Gerada et al., 2020). Immune suppression or exposure to various environmental stimuli has been shown to activate latent herpesvirus to undergo lytic replication (Aneja and Yan, 2017; Forte et al., 2020). Latently infected herpesviruses are periodically activated, reentering the active replication to produce large numbers of infectious virions that can be transmitted to new hosts (Broussard and Damania, 2020). The balance between the lytic and latent phases is an important strategy for herpesvirus survival *in vivo* and results in lifelong survival of the virus and host-to-host the transmission from host to host (Aneja and Yan, 2017).

**FIGURE 1:**
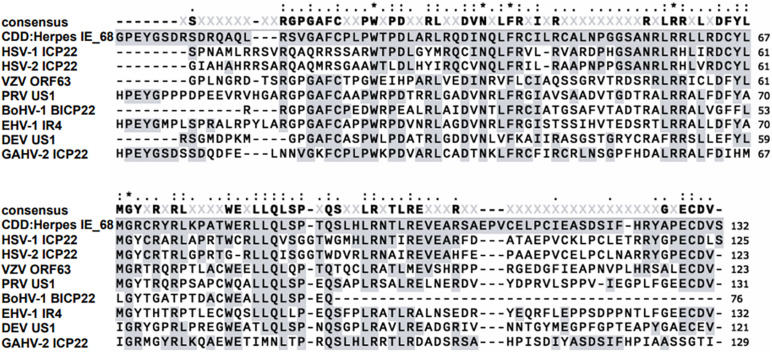
Sequence alignment of the conserved IE-68 domain of representative human herpesviruses.

Human herpesviruses are the causative agents of many common diseases, including chickenpox, shingles, mononucleosis, cold sores, and genital herpes (Kimberlin and Rouse, 2004). Eight herpesviruses are known pathogens of humans: herpes simplex virus (HSV) 1 and 2 and varicella zoster virus (VZV) are α-herpesviruses; cytomegalovirus and human herpesvirus (HHV) 6 and 7 are β-herpesviruses, whereas Epstein–Barr virus and HHV8 are γ-herpesviruses infecting humans (Carter et al., 2007; Chayavichitsilp et al., 2009). ICP22 are homologs conserved in all α-herpesviruses; these proteins contain an IE-68 domain conserved in herpesviruses (shown in Figure 1) and are expressed from an immediate–early (IE) gene during the replication cycle of HSV-1/2 and VZV. They have been suggested to play various roles in the viral life cycle that are important for efficient viral replication, latency, and reactivation (summarized in Table 1). HSV-1 ICP22 can generally regulate viral and host gene transcription by changing the phosphorylation status of host RNA polymerase II (RNA pol II) (Bastian and Rice, 2009; Lin et al., 2010; Zaborowska et al., 2014; Fox et al., 2017) and can also facilitate the nuclear egress complex (NEC) accurately locate to the nuclear membrane to promote nuclear budding (Liu et al., 2015), whereas VZV ORF63regulates VZV transcription by destabilizing preinitiation complex (PIC) formation (Di Valentin et al., 2005). In addition, both ICP22 and ORF63 transcripts have been suggested to be important in latency (Kennedy et al., 2015; Depledge et al., 2018a; Matundan et al., 2019; Ouwendijk et al., 2020; Tormanen et al., 2020). By contrast, there are few reports of the participation of ICP22 in the lytic and latent infection of HSV-2. This protein is best known for its function as an E3 ubiquitin ligase involved in the protein ubiquitination pathway. This pathway is related to protein modification and plays a role in the inhibition of innate immunity by preventing interferon (IFN)–mediated signaling. Also, it induces the ubiquitination and degradation of host STAT1, STAT2, and IRF9, which results in the blockade of ISGF3 nuclear translocation (Zhang et al., 2015, 2020). Thus, considering that the main focus of this review is a discussion of the various functions and mechanisms of ICP22/ORF63 during the lytic and latent phases of human herpesvirus infection, we omit HSV-2 ICP22 and focus particularly on the HSV-1 ICP22 and VZV ORF63, which are involved in viral transcription, nuclear egress, and latency and participate in interplay with viral and cellular proteins. We believe that illustrating the functions and underlying mechanisms of ICP22/ORF63 during the human herpesvirus life cycle will help to elucidate the pathogenesis of the related diseases and contribute to the development of new antiviral drugs and vaccines.

**TABLE 1:** Viral and cellular factors that interact with ICP22/ORF63 proteins during the human herpesvirus life cycle.

Process of in the herpesvirus life cycle	ICP22/ORF63 protein	Interaction partners	Functional consequence	References
Transcription	HSV-1 ICP22	VP16	Recruits P-TEFb to promote transcriptional elongation, offsetting the inhibition of transcription by ICP22	Guo et al., 2012
		UL13	Phosphorylates ICP22 and functions as a switch to alter the activity of ICP22, thereby mediating the induction of RNA pol II	Fraser and Rice, 2007
		P-TEFb (cdk9, cyclin T1)	Triggers the loss of Ser-2 phosphorylation in the RNA pol II C-terminal domain, thus regulating viral and host gene transcription	Durand et al., 2005; Durand and Roizman, 2008; Guo et al., 2012; Zaborowska et al., 2014
		Spt5, Spt6, FACT (Spt16, SSRP1)	Is recruited to the HSV-1 genome by ICP22 to facilitate the elongation of viral transcripts by ensuring a looser chromatin structure on viral gene bodies	Fox et al., 2017
	VZV ORF63	TFIIH, TFIIE, RNA pol II	Mediates destabilization of the PIC and the removal of several general transcription factors from certain promoters	Lynch et al., 2002; Di Valentin et al., 2005
Nuclear egress	HSV-1 ICP22	UL31, UL34, UL47, Us3, p32	Forms a complex with ICP22 and participates in the process of nuclear egress	Liu et al., 2015
Latency	HSV-1 ICP22	Cluster of differentiation 80 (CD80)	Can bind to the CD80 promoter and inhibit its activity to enhance latency and reactivation	Matundan et al., 2019; Tormanen et al., 2020
		ICP0, ICP27, ICP47	Increases the activity of CD80 but cannot offset ICP22-mediated inhibition of CD80	Tormanen et al., 2020
	VZV ORF63	Anti–silencing factor 1	Contributes to the establishment or maintaining the latency of VZV in the trigeminal ganglia (TG)	Ambagala et al., 2009
		VZV latency-associated transcript	Is coexpressed in human TG, thereby acting as an initiator of viral gene transcription during reactivation	Depledge et al., 2018a; Ouwendijk et al., 2020

## Roles of ICP22/ORF63 Proteins in Viral and Cellular Transcriptional Regulation

ICP22/ORF63 proteins first attracted attention in virology because of its function as transcriptional regulators of cellular and viral mRNAs. Viruses lack the basic machinery for replication and must hijack the relevant functions of host cells to complete the viral replication cycle in order to produce progeny virions. Like most nuclear-replicating DNA viruses, herpesviruses use the cellular enzyme RNA pol II for the transcription of viral genes (Gruffat et al., 2016). RNA pol II–mediated transcription consists of three steps: initiation, elongation, and termination (Hahn, 2004; Liu et al., 2013; Schier and Taatjes, 2020). During initiation, RNA pol II and general transcription factors (GTFs) bind to gene promoter regions and initiate the synthesis of nascent RNA (Krishnamurthy and Hampsey, 2009; Sainsbury et al., 2015; Haberle and Stark, 2018); during elongation, RNA pol II moves along the template strand to the gene body for extension of nascent RNA (Sims et al., 2004; Chen et al., 2018a); and during termination, upon completion of the processing of the nascent RNA transcript, RNA pol II and the mature RNA are released from the template DNA (Wahle and Keller, 1996; Richard and Manley, 2009; Porrua et al., 2016). Each of these steps requires the involvement of RNA pol II and its regulation by different proteins to ensure the correct transcription and expression of genes (Peck et al., 2019). As discussed below, ICP22/ORF63 are key proteins that participate in each step of RNA pol II–mediated viral gene transcription.

### VZV ORF63 Destabilizes the PIC at Certain Promoters

**FIGURE 2:**
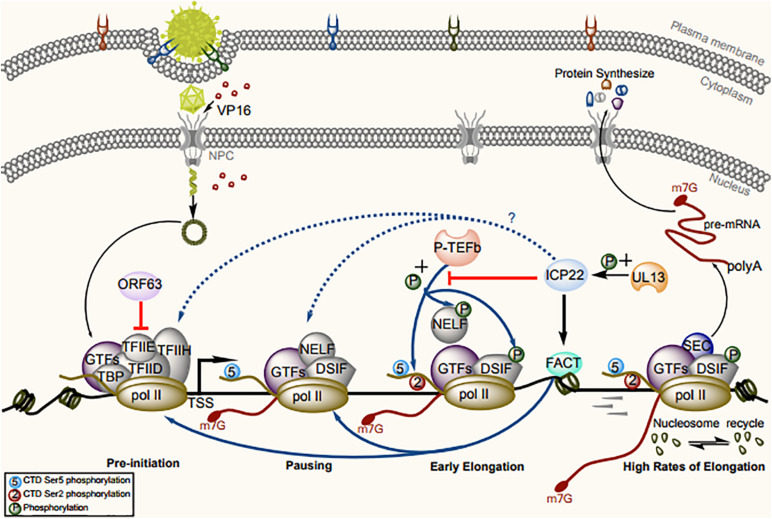
Model for the regulation of RNA pol II–mediated transcription by ICP22/ORF63 and cofactors. Assembly and initiation of the PIC are the first steps in transcription. RNA pol II and GTFs are recruited to the accessible chromatin architecture around the promoter. The CTD of RNA pol II is generally unmodified in the PIC. VZV ORF63 can sequester RNA pol II and TFIIE from promoters to disrupt PIC assembly. After synthesis, a 20- to 60-nt RNA transcript, the pause-inducing factors DSIF and NELF bind the early elongation complex, inhibiting further transcription. The GTF TFIIH complex contains the kinase CDK7, which phosphorylates the RNA pol II CTD at Ser-5, resulting in recruitment of the capping enzyme complex to add a 5′ cap to the nascent RNA. The release of paused RNA pol II is triggered after phosphorylation of RNA pol II CTD Ser-2 and pause-inducing factors by P-TEFb, to dissociate NELF and promote productive elongation. At this stage, ICP22 can trigger loss of phosphorylation of Ser-2 in the RNA pol II CTD by interacting with P-TEFb, and VP16 can recruit P-TEFb into the viral promoter region, thus offsetting ICP22-mediated inhibition of transcription. During elongation, ICP22 can interact with the FACT complex to promote the production of viral progeny by ensuring looser chromatin architecture on viral gene bodies.

Transcription initiation is the main step in the regulation of gene expression (Haberle and Stark, 2018). RNA pol II requires the proper assembly of at least eight cellular GTFs: TFIIA, TFIIB, TFIID, TFIIE, TFIIF, TFIIH, RNA pol II, and mediator, to assemble the PICs on core promoter elements (Hahn, 2004; Krishnamurthy and Hampsey, 2009; Sikorski and Buratowski, 2009; Hahn and Young, 2011; Spitz and Furlong, 2012; Nogales et al., 2017; Haberle and Stark, 2018). Once all components precisely assemble and bind, the PIC can “open” the promoter DNA to initiate transcription (Schier and Taatjes, 2020). Transient-transfection assays showed that VZV ORF63 acts as a transcriptional repressor of all genes with a TATA box sequence in the promoter. This protein was found to be immunoprecipitated with TFIIH and, to a lesser extent, TFIIE and RNA pol II, resulting in instability of the PIC and dissociation of GTFs from the promoter (Figure 2). The central and carboxy-terminal domains of IE63 (ORF63 encoded protein) are important for these effects. The transcriptional inhibitory ability of ORF63 has been confirmed in two different cell lines—a Vero cell line with lytic VZV infection and an ND7 cell line with maintained latent VZV infection—demonstrating that its inhibitory ability is independent of the cell type and infection pattern (Di Valentin et al., 2005).

### HSV-1 ICP22 Generally Regulates Viral and Host Gene Transcription by Changing the Phosphorylation Status of Host RNA Pol II

The greatest difference between RNA pol II, RNA pol I, and RNA pol III is the conserved C-terminal domain (CTD) of Rpb1, which is the largest subunit of RNA pol II (Cramer et al., 2008). The CTD contains a conserved heptapeptide repeat, Tyr1-Ser2-Pro3-Thr4-Ser5-Pro6-Ser7 (Chapman et al., 2008; Egloff and Murphy, 2008). The number of repeats varies across species (Chapman et al., 2008; Eick and Geyer, 2013). The CTD can be used as a platform to recruit transcription factors to RNA pol II, which plays an important role in gene transcriptional regulation (Komarnitsky et al., 2000; Buratowski, 2003; Egloff and Murphy, 2008; Zaborowska et al., 2016; Harlen and Churchman, 2017; Engel et al., 2018). Five amino acid residues in the heptapeptide repeat can be phosphorylated (Hsin et al., 2014), and the dynamic changes in the phosphorylation status during transcription are closely related to the recruitment of specific regulatory proteins (Gomes et al., 2006; Peck et al., 2019). The regulation of transcription elongation is strongly linked to the CTD phosphorylation status (Cadena and Dahmus, 1987; Payne et al., 1989; Harlen and Churchman, 2017). Phosphorylation occurs mainly at Ser-2 and Ser-5 during transcription (McCracken et al., 1997; Schroeder et al., 2000; Licatalosi et al., 2002; Kim et al., 2009; Harlen and Churchman, 2017; Nemec et al., 2019). Pol II pausing is induced when RNA pol II binds to negative elongation factor (NELF) and 5,6-dichloro-1-β-D-ribofuranosylbenzimidazole sensitivity-inducing factor (DSIF), which is a heterodimer formed by SPT4 and SPT5 (Ping and Rana, 2001; Adelman and Lis, 2012; Natarajan et al., 2013; Jonkers and Lis, 2015; Core and Adelman, 2019; Aoi et al., 2020). RNA pol II recruits the positive transcription elongation factor B complex (Peterlin and Price, 2006; Ni et al., 2008; Zhou et al., 2012; Franco et al., 2018), in which cyclin-dependent kinase 9 (CDK9) phosphorylates NELF and DSIF to dissociate NELF from the transcriptional pausing complex and convert DSIF into a positive transcription elongation factor, promoting pausing release and elongation (Peterlin and Price, 2006; Paparidis et al., 2017).

HSV-1 ICP22 encodes an embedded protein, US1.5; which is initiated from methionine 147 of ICP22 and colinear with the remaining portion of that protein, and the translation of each protein is driven by independent promoters (Carter and Roizman, 1996). US1 encodes two sets of functions, one in the amino terminus unique to ICP22 and one shared by ICP22 and US1.5 (Ogle and Roizman, 1999), and these functions are required for efficient HSV-1 replication in most cell types *in vitro* and *in vivo* (Orlando et al., 2006). Either protein can functionally enhance the expression of late (L) viral proteins in cell culture and inhibit ICP0-mediated gene expression in transient-transfection assays. However, researchers have not defined a unique function for US1.5 or demonstrated a defect in HSV-1 replication in its absence, which suggests that the viral requirement for US1.5 is conditional and is enhanced only under certain circumstances during the HSV-1 life cycle, further suggesting that US1.5 can function as a biological backup for ICP22 should ribosome read-through be blocked at the translation initiation codon of ICP22 (Bastian and Rice, 2008). Follow-up studies showed that residues 193–256 of ICP22 can interact with CDK9 without affecting its recruitment and inhibit the phosphorylation of Ser-2 in the RNA pol II CTD (Durand et al., 2005; Justyna et al., 2014) but do not directly interact with RNA pol II (Rice et al., 1995; Fraser and Rice, 2005; Guo et al., 2012; Mostafa and Davido, 2013; Rice and Davido, 2013; Figure 2). Expression of the HSV-1 ICP22-related protein US1.5 also triggers loss of Ser-2 phosphorylation in RNA pol II in transfected cells (Fraser and Rice, 2007). Ser-2 phosphorylation induced by CDK9 is necessary for HSV-1 transcription and replication. When CDK9 activity is inhibited, the number of progeny and the expression level of late genes decrease, and these events adversely affect the formation of the viral replication compartment (Durand and Roizman, 2008). In addition, the functions of ICP22 as a general transcriptional regulator can also be regulated by other viral proteins. Previous studies have shown that ICP22-mediated loss of Ser-2 phosphorylation in RNA pol II does not depend on the HSV-1 protein kinase UL13, which can directly or indirectly affect ICP22 and US1.5 phosphorylation (Purves and Roizman, 1992; Asai et al., 2007; Fraser and Rice, 2007). However, in some experiments, the loss of phosphorylation of RNA pol II Ser-2 in the UL13-deficient strain was accelerated compared to that in the wild-type strain, suggesting that the newly expressed UL13 may alter the activity of ICP22, thereby affecting its ability to mediate the phosphorylation of Ser-2 in RNA pol II (Long et al., 1999; Fraser and Rice, 2007). Moreover, when the recruitment of P-TEFb is inhibited by ICP22, the viral transactivation protein VP16 can recruit P-TEFb to promoter region, thus offsetting ICP22-mediated inhibition of transcription (Guo et al., 2012). As P-TEFb is isolated mostly in the 7SK small nuclear ribonucleoprotein (snRNP) complex in an inactive state, it is presumed that VP16 can cause the dissociation of P-TEFb from the 7SK snRNP and functionally activate P-TEFb (Li et al., 2017). However, researchers did not observe loss of RNA pol II Ser-2 phosphorylation in VZV ORF63, the homolog of ICP22 in HSV-1, suggesting that this function is not conserved between HSV-1 and VZV (Fraser and Rice, 2007).

### HSV-1 ICP22 Interacts With FACT to Influence Transcription-Coupled Histone Modification

Facilitates chromatin transcription (FACT), which can the ability to destabilize nucleosomes, has long been considered as a transcriptional elongation factor that promotes RNA pol II progression on chromatin templates (LeRoy et al., 1998; Mason and Struhl, 2003; Tettey et al., 2019; Formosa and Winston, 2020). The FACT complex is a heterodimer consisting of two distinct subunits Spt16 and SSRP1 (Orphanides et al., 1999; Formosa, 2012; Zhou et al., 2020) and participates in the assembly and disassembly of nucleosomes encountered by the transcribing polymerase (Belotserkovskaya et al., 2003; Liu et al., 2020). DNA replication requires rapid assembly of large numbers of nucleosomes to form chromatin (Reinberg and Sims, 2006; Formosa, 2012). FACT may be associated with the establishment and maintenance of loose chromatin structure in HSV-1 genomic DNA during replication (Placek and Berger, 2010). Recent studies have suggested that FACT plays a broad role in maintaining chromatin structure and RNA pol II pausing at promoter-proximal pause sites (Tettey et al., 2019). In addition, multiple lines of evidence suggest that FACT also plays a role in transcription initiation. Overexpression subunits of FACT or deficiency of its activity lead to inadequate disinhibition of a transposon-associated promoter in yeast (Clark-Adams et al., 1988; Malone et al., 1991), whereas mutation of FACT results in increased gene transcription of some genes (Pelechano et al., 2009; Feng et al., 2016; Pathak et al., 2018), suggesting a global role of FACT in blocking improper transcription initiation by maintaining chromatin in a particular form (Formosa and Winston, 2020). In accord with previous results (Pathak et al., 2018), depletion of FACT greatly reduces PIC assembly and transcription *in vivo* (Petrenko et al., 2019). FACT may play an important role in the maintenance or crossing of chromatin barriers outside transcription units prior to the initiation or extension stages of transcription (Belotserkovskaya et al., 2003; Schwabish and Struhl, 2004; Hsieh et al., 2013; Chen et al., 2018b; Formosa and Winston, 2020). After the promoter escapes, RNA pol II must overcome the disruption of transcription induced by nucleosomes, whereas FACT can reshape nucleosomes and reduce this disruption of transcription. In the presence of FACT, the paused state of RNA pol II can be maintained (Bondarenkoa et al., 2015). When FACT is depleted, RNA pol II returns to its elongation activity (Tettey et al., 2019). During HSV-1 transcription, ICP22 interacts with the FACT complex, resulting in altered localization of FACT in the nucleus and the recruitment of two other transcriptional elongation factors encoded in the viral genome (Spt5 and Spt6), thus maintaining a looser chromatin structure in the viral genome and promoting the production of progeny virions (Fox et al., 2017). Whether ICP22 inhibits CDK9 activity or recruits FACT to the transcriptional pause site, we can conclude that ICP22 plays a role during the pausing of RNA pol II, but the effects of the two modes of action are different. The former mode of transcriptional pausing enables FACT to help RNA pol II overcome obstacles and transition transcription from pausing to elongation (Figure 2). Although the mechanism is unclear, the interaction between ICP22 and FACT may be highly important for inhibition or activation at specific stages of the viral life cycle, and this possibility should be the focus of further study.

## ICP22 Promotes Primary Envelopment by Interacting With the NEC

**FIGURE 3:**
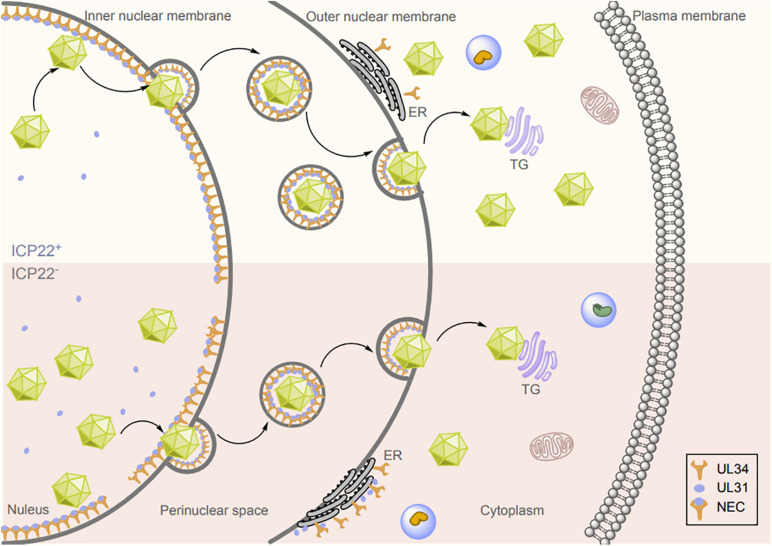
Effects of ICP22 on herpesvirus nuclear budding. Herpesvirus nucleocapsid assembly is completed in the nucleus, and the nucleocapsid is then translocated by budding from the INM to the perinuclear region via the NEC, where it fuses with the ONM to complete the budding and release process. ICP22 promotes HSV-1 nuclear budding by interacting with and regulating UL31 and UL34.

During herpesvirus infections, DNA replication and nucleocapsid formation occur in the nucleus of the host cell, whereas further maturation of virions occurs in the cytoplasm. Therefore, the viral capsid must pass through the nuclear envelope to enter the final maturation compartment (Johnson and Baines, 2011; Mettenleiter et al., 2013a; Lv et al., 2019). Transit of the viral nucleocapsid across the nuclear membrane is the first step in early virion maturation. Therefore, an important mechanism by which the viral nucleocapsid traverses the nuclear membrane is the destruction of the nuclear membrane structure (Johnson and Baines, 2011). Nuclear budding of the herpesvirus capsid through the nuclear membrane is mediated by the NEC (Bigalke and Heldwein, 2017). In HSV-1, the NEC is a heterodimer composed of the a type II membrane protein UL34 (Ott et al., 2011) and UL31, and the absence of UL34 or UL31 abolishes nuclear budding and affects the generation of infectious progeny (Chang et al., 1997; Roller et al., 2000; Matundan and Ghiasi, 2018; Takeshima et al., 2019). UL34 is localized mainly on the inner nuclear membrane (INM), outer nuclear membrane (ONM), and endoplasmic reticulum (ER) (Ott et al., 2011; Schuster et al., 2012), whereas UL31 is a nucleophosphoprotein that contains nuclear localization signals and is not membrane anchored (Chang and Roizman, 1993; Arii et al., 2019). When UL31 and UL34 are coexpressed, UL31 can be relocalized to the surface of the INM through the interaction with UL34, and the N-terminal domain of pUL31 was required to prevent their premature interaction in the cytoplasm. Then, the virion and cellular kinases are relocalized, causing some proteins to exert phosphorylation activity and locally degrade the nuclear membrane, after which the capsid in the nucleus can reach the budding site on the INM (Granzow et al., 2001; Mettenleiter, 2004; Roller et al., 2010; Mettenleiter et al., 2013b; Funk et al., 2015; Lorenz et al., 2015a; Yadav et al., 2017). In the absence of assistance from other viral proteins, UL31 and UL34 can guide the formation of vesicles from the INM and promote nuclear budding *in vivo* (Klupp et al., 2007; Desai et al., 2012; Hagen et al., 2015); further *in vitro* analysis suggested that the recombinant HSV-1 pUL31 and pUL34 can drive membrane budding and scission of vesicles (Bigalke et al., 2014; Lorenz et al., 2015b). ICP22 has been reported to form a complex with UL31, UL34, UL47, and US3 after HSV-1 infection, resulting in colocalization of ICP22, UL31, and UL34 on the nuclear membrane in infected cells (Mou et al., 2009; Liu et al., 2014; Maruzuru et al., 2014). These proteins have a non-negligible role in the nuclear egress of virions. Therefore, the primary envelopment efficiency of HSV-1 caused by ICP22 deletion or mutation may be altered through its interaction with and regulation of UL31 and UL34. During this process, UL31 guides the recruitment and anchoring of ICP22 to the nuclear membrane, and ICP22 promotes the correct localization of UL31 and UL34 at the nuclear membrane. Indeed, deletion or mutation of ICP22 caused mislocalization of UL31 and UL34 in the ER (Liu et al., 2015). However, although enveloped virions were still detected in the cytoplasm and on the cell surface after ICP22 deletion, the number of enveloped viruses in the perinuclear region was significantly reduced (Figure 3). These results suggest that ICP22 plays a regulatory role in but is not essential for the primary envelopment of HSV-1 virions.

## Roles of ICP22/ORF63 in Viral Latency and Reactivation

The balance between the life cycles of lytic and latent herpesvirus infection is a complex and finely tuned process that allows herpesvirus to persist and spread throughout the life of the host (Broussard and Damania, 2020). Lytic infection is critical for host-to-host transmission and pathogenicity of herpesvirus. During latency, the virus enters a state of limited gene expression and does not replicate. In the trigeminal ganglia (TG) of humans, HSV-1 (Yan et al., 2020) and VZV (Depledge et al., 2018a) genomic DNA is not replicated, but exists as continuous (circular) episomes or genome concatemers in latently infected cells, and only limited viral gene transcription occurs to maintain latent infection and evade host immune surveillance (Efstathiou et al., 1986; Clarke et al., 1995; Gary et al., 2006; Muylaert et al., 2011; Nicoll et al., 2012; Depledge et al., 2018a,b). The expression of genes during latency varies considerably among herpesviruses. Latency-associated transcripts (LATs) map antisense to ICP0/RL2 within the long repeat (RL) (Stevens and Cook, 1971; Spivack and Fraser, 1987; Fareed and Spivack, 1994; Wu et al., 1996; Nicoll et al., 2012) and are the only viral transcripts generally detected during HSV-1 latency (Kennedy et al., 2015). However, VZV latency in the TG of humans is characterized by expression of VZV latency-associated transcript (VLT) and the ORF63 transcript (Depledge et al., 2018a). VLT encodes the antisense transcript of ORF61 and is considered a homolog of the LATs encoded by all other well-studied neurotropic α-herpesviruses (Depledge et al., 2018b; Baird et al., 2019). However, VZV appears to be the only α-herpesvirus that has been analyzed in detail and consistently expresses an additional latency transcript, ORF63, in the TG of humans (Depledge et al., 2018b).

### ORF63 Plays an Important Role in the Establishment of VZV Latency

Before the discovery of VLT, ORF63 was the first VZV gene shown to be critical for the establishment of latency (Cohrs et al., 1996; Kennedy et al., 1999; Kennedy et al., 2000; Cohen et al., 2004). VZV with ORF63 deletion exhibits impaired replication in melanoma cells and fibroblasts and impaired latency in rodents, but its ability to infect ganglia of the rodents is not impaired (Sadzot-Delvaux et al., 1995; Kennedy et al., 2001; Sommer et al., 2001). During lytic infection of VZV, IE-63 (the protein encoded by ORF63) is detected mainly in the cytoplasm of latently infected sensory neurons (Mahalingam et al., 1996; Lungu et al., 1998), but is expressed mainly in the nucleus during lytic infection *in vitro* (Debrus et al., 1995; Stevenson et al., 1996; Ambagala et al., 2009). The genome copy numbers and numbers of cotton rats that could establish latent VZV infection were lower for infection with an ORF63 gene deletion strain than for infection with the parental strain (Cohen et al., 2004). Similar studies showed that when animals were inoculated with the wild-type, ORF63 mutant, and revertant strains, the copy numbers in dorsal root ganglia and the frequency of latent infection were the lowest in ORF63 mutant-infected animals. Thus, ORF63 is the key to establishing latent infection but is not necessary for VZV entry into the ganglia (Cohen et al., 2004). Further identification of ORF63 domains revealed that the replacement of five serine or threonine phosphorylation sites in the last 108 amino acids of ORF63 with alanines resulted in impaired viral replication and latency establishment *in vitro*. This finding suggests that a region of ORF63 important for VZV replication *in vitro* is required for efficient establishment of latency (Cohen et al., 2005).

In addition, IE63 was found to be able to lock part of anti–silencing factor 1 (ASF1) into the cytoplasm in a model of latent VZV infection of enteric neurons in guinea pigs (Ambagala et al., 2009). ASF1, a member of the H3/H4 family of histone chaperones, is a nucleosome assembly factor that participates in a variety of cellular functions, including DNA replication, gene transcription, and the cellular response to DNA damage by histone removal from and deposition onto DNA (Donham et al., 2011; Zhang et al., 2018, 2019; Cote et al., 2019). In VZV-infected and IE-63–transfected cells, ASF1 colocalized and immunoprecipitated with IE-63. In addition, colocalization of IE-63 and ASF1 was observed in intestinal neurons with lytic and latent VZV infection (Ambagala et al., 2009). Infection with site mutants (such as ROKA63-ACCI and ROKA63-5M) impaired the interaction of IE-63 and ASF1, and infection with a mutant with complete deletion of the IE-63 gene (ROKA63D) led to impaired establishment of VZV latency in rodents (Cohen et al., 2004, 2005). These results suggest that the interaction between IE-63 and ASF1 may contribute to the establishment or maintenance of latent VZV infection.

### ICP22 Enhances Latency and Reactivation

HSV-1 evades clearance by the host immune system through a variety of mechanisms to establish lifelong latent infection (Kurt-Jones et al., 2017; Lin and Zheng, 2019; Tognarelli et al., 2019; Zhu and Zheng, 2020). For example, HSV-1 LAT plays a vital role in generating dysfunctional T-cell responses in the TG of ocular infected HSV-1 mice and helps the virus resist host cell apoptosis by inhibiting the type I IFN signaling pathway, thus contributing to the establishment and activation of latent HSV-1 infection (Perng et al., 2000; Branco and Fraser, 2005; Allen et al., 2011; Tormanen et al., 2019; Jaggi et al., 2020). LAT-containing neurons are occasionally surrounded by CD8^+^ T cells (Theil et al., 2003; Verjans et al., 2007) that are primed in the periphery (Held et al., 2011) and LAT functions in part to protect neurons against granzyme B–induced apoptosis (Allen et al., 2011; Jiang et al., 2011). A recent study reported the novel finding that ICP22 can downregulate host costimulatory molecular cluster of differentiation 80 (CD80) after ocular HSV-1 infection to promote host immune evasion of HSV-1 (Matundan et al., 2019). CD80, as a stimulator of T cells, plays a key role in the activation and proliferation of T cells and can be expressed after ocular infection, thus triggering the immune response and causing corneal scarring (Lanier et al., 1995; Bugeon et al., 2006; Jaggi et al., 2020; Tormanen et al., 2020). Compared with wild-type virus-infected cells, infection of 293 cells with ICP22-deficient viral strain resulted in increased CD80 promoter activity, decreased virus replication and latency, and delayed reactivation from latency (Matundan and Ghiasi, 2019). *In vivo* experiments showed that the level of the host immune response decreased after ICP22-mediated inhibition of CD80. Mutation of 34 or 116 amino acids in the ICP22 sequence affected the replication and pathogenicity of the virus but did not release the binding of ICP22 to CD80, and the immune response was still suppressed in mice with corneal infection (Matundan et al., 2019). Relevant studies showed that ICP0, ICP27, and ICP47 can increase the activity of the CD80 promoter, but none can offset ICP22-mediated inhibition of CD80 (Lv et al., 2018). A transient reduction in the dendritic cell population through downregulation of CD80 by ICP22 could explain the enhanced latency and reactivation (Tormanen et al., 2020).

### VLT-ORF63 Fusion Transcripts Are Potentially Involved in the Transition From Latency to Lytic VZV Infection

Using a highly sensitive enriched RNA sequencing (RNA-Seq) enrichment method, researchers found no evidence for the expression of VZV mRNA other than VLT and ORF63 during latent infection (Depledge et al., 2018a). VLT was defined as a novel 496-nucleotide multiexon transcript transcribed antisense to ORF61 via ultradeep virus-enriched RNA-Seq of latently infected human TG (Depledge et al., 2018a). Further reverse transcriptase–polymerase chain reaction (PCR) and quantitative PCR analyses showed that the latent VZV also transcribes the lytic ORF63 gene at lower levels relative to VLT in a subset of latently infected TG, independent of the latent viral DNA load (Depledge et al., 2018a). Although the expression levels were different, the expression levels of VLT and ORF63 transcripts were significantly correlated, suggesting that they coregulate expression during latent VZV infection (Ouwendijk et al., 2012; Depledge et al., 2018a). This apparent expression of two distinct viral transcripts during latency is unique among well-studied α-herpesviruses, suggesting that these two transcripts and/or their encoded proteins play an important role in the latency and reactivation of VZV (Ouwendijk et al., 2020). In the human induced pluripotent stem cell–derived sensory neuron (HSN) latency model *in vitro*, the results of coexpression of VLT and two VLT-ORF63 fusion products, VLT63-1 and VLT63-2, showed that the encoded pVLT-ORF63 fusion protein can act as an initiator of VZV reactivation in infected human TG, providing new insights into the mechanism by which VZV establishes latency and reactivation (Ouwendijk et al., 2020). Moreover, ectopic VLT63-1 induces lytic transcription of viral genes in HSN models of latent VZV infection, suggesting that the pVLT-ORF63 fusion protein, not the corresponding transcript, induces transcriptional activation of VZV genes. However, pORF63 did not induce the transcription of any IE, early or late gene, except for the IE promiscuous transactivator pORF61 and the early (E) transcript RNA 16-1 (Moriuchi et al., 1993). These results indicated that gene transcription during VZV reactivation is initiated through VLT-ORF63 transcription/pVLT-ORF63 translation (Ouwendijk et al., 2020). However, whether VLT and ORF63 RNA transcripts are produced by the same or distinct populations of neurons remains unclear, as human TG are composed of dissimilar subtypes of neurons, and how these transcripts may affect ability of VZV to reactivate from latent in neurons is unknown (Depledge et al., 2018b). Thus, more studies such as *in situ* analyses are required to identify the types of neurons in which coexpression of VLT and ORF63 RNA and the latent VZV genome are present. By contrast, no initiator that drives viral gene expression during reactivation has been reported in HSV-1 (Wilson and Mohr, 2012), suggesting that the mechanisms controlling latent infection and reactivation may differ between HSV and VZV.

## Summary

Herpesviruses are extremely successful parasites that have evolved over millions of years to develop a variety of mechanisms to coexist with their hosts and maintain host-to-host transmission and lifelong infection by regulating their life cycles (Adler et al., 2017; Renner and Szpara, 2018). Inhibition of viral replication treats lytic herpesvirus infections but does not cure latent infection (Skoreński and Sieńczyk, 2014; Gershon et al., 2015; van Diemen and Lebbink, 2017; Andrei et al., 2019). An in-depth understanding of the molecular mechanism by which viral proteins regulate the life cycle will offer new insights into the treatment of herpesvirus infections via the destruction of key proteins in the herpesvirus life cycle, thus providing new strategies for the treatment of herpesvirus infections and the resulting diseases (Broussard and Damania, 2020). In this article, we summarized the recent findings on the involvement of the ICP22/ORF63 proteins in the viral life cycle. The broad interplay of HSV-1 ICP22 or VZV ORF63 with cellular and viral proteins contributes to various processes in the herpesvirus life cycle, including transcription, nuclear egress, and establishment of latency and reactivation, which are critical to herpesvirus survival. Any drug that interferes with these steps can disrupt the viral life cycle as a therapeutic strategy for herpesvirus infections. Further research into the processes and underlying mechanisms of the viral life cycle will help to achieve this goal. However, there are very few reports on ICP22/ORF63 homologs in animal herpesviruses, such as duck enteritis virus, Marek disease virus, equine herpesvirus 1, bovine herpesvirus 1, and pseudorabies virus, and researchers must devote more attention to these viruses in the future. The multifaceted roles and complex mechanisms by which of ICP22/ORF63 proteins regulate the life cycle of herpesviruses offer both a foundation and a challenge for our understanding of the mechanisms and for the development of various potential treatment options. As we learn more about these proteins, we can identify the protocols with the greatest potential for herpesvirus treatment and vaccine development.

## Author Contributions

MW, RJ, SC, QiaoY, DZ, ML, XZ, SZ, JH, XO, SM, QG, DS, and BT provided ideas contributing to the structure of this manuscript. AC modified the manuscript. All authors listed contributed to the completion of the manuscript, reviewed and approved the final manuscript. YW and QiaoY contributed to the design and writing of the article.

## Conflict of Interest

The authors declare that the research was conducted in the absence of any commercial or financial relationships that could be construed as a potential conflict of interest.
